# The Environmental Burden and Sustainability Challenges of Clear Aligners: Implications for Public Health, Practice, Policy, and Future Research—A Scoping Review

**DOI:** 10.1155/ijod/2681058

**Published:** 2026-08-30

**Authors:** Sakshi Sakshi, Asavari Desai, Parul Dasson Bajaj

**Affiliations:** ^1^ Department of Orthodontics and Dentofacial Orthopaedics, Manipal College of Dental Sciences Mangalore, Manipal Academy of Higher Education, Manipal, India, manipal.edu; ^2^ Department of Public Health Dentistry, Manipal College of Dental Sciences Mangalore, Manipal Academy of Higher Education, Manipal, India, manipal.edu

**Keywords:** clear aligners, climate action, environment, leaching, microplastics, public health, sustainability

## Abstract

**Background:**

Malocclusion is a major global oral health concern and has driven the rapid adoption of clear aligner therapy (CAT) as an esthetic and removable alternative to conventional fixed appliances. However, the widespread use of CAT has raised important concerns regarding its environmental footprint and potential public health implications, including waste generation, chemical leaching, and the release of microplastics. This scoping review aimed to systematically synthesize the available evidence on the environmental and public health implications of CAT and identify key areas in practice, research, and policy to advance toward sustainable oral healthcare services in alignment with the World Health Organization’s Global Oral Health Action Plan 2023–2030.

**Methods:**

This scoping review was conducted in accordance with Arksey and O’Malley’s framework and reported in line with PRISMA‐ScR guidelines. Eligible sources were identified through a search across four databases, namely, PubMed, Scopus, Web of Science, and Embase, to identify literature addressing environmental or public health aspects of clear aligners, published in English, either in print or online ahead of print, between January 2005 and December 2025. Data charting was conducted, followed by qualitative analysis using ATLAS.ti to identify the key themes emerging from included sources relating to the research question.

**Results:**

A total of 61 sources were included. The major themes which emerged from this scoping review included material toxicity and chemical exposure, microplastic and nanoplastic release, environmental burden of CAT, technological transitions in the form of 3D/4D aligners, strategies toward sustainability and governance, regulation, and research gaps.

**Conclusions:**

CAT must be recognized not only as an orthodontic innovation but also as an emerging public health and sustainability challenge, highlighting the need for stronger evidence, safer materials, improved waste management, and clearer regulations to align future orthodontic care with Sustainable Development Goals 3 (Good Health and Well‐being) and 13 (Climate Action).

## 1. Introduction

Malocclusion poses a major oral health burden, affecting nearly one in two children and adolescents worldwide, with reported prevalence ranging from 48% to 81% across different regions [1]. This high prevalence has driven substantial demand for orthodontic care, where clear aligner therapy (CAT) has emerged as a transformative innovation in contemporary practice. By offering an esthetic, removable alternative to conventional fixed appliances, CAT has achieved rapid and widespread global adoption over a relatively short period of time [2, 3]. It is estimated that more than 14 million patients have undergone treatment using clear aligners, resulting in the production of hundreds of millions of plastic models and aligners [4].

The scale of this adoption raises important concerns regarding its material and environmental footprint. Based on available estimates, the treatment of ~14 million patients corresponds to the production of nearly 728 million aligners, and considering the average weight of 2 g per aligner, this equates to ~1456 tons of plastic generated from aligners alone. However, this figure underestimates the environmental burden of aligners as it does not account for the production and distribution processes. The thermoforming process in aligner production yields substantial waste, with nearly three‐quarters to four‐fifths of the aligner sheet discarded during production. Furthermore, the production process involves the use of 3D‐printed models, which contribute additional plastic waste, along with the packaging materials and containers required for distribution. Collectively, these factors highlight the significant and often underappreciated environmental burden associated with the large‐scale adoption of CAT [5].

Clear plastic aligners are typically composed of polyethylene terephthalate (PET), PET glycol‐modified (PET‐G), and thermoplastic polyurethane (TPU), among other petroleum‐based polymers [2, 6]. While these materials possess the necessary mechanical properties, their nonbiodegradable nature raises a significant environmental concern. Furthermore, the largely single‐use nature of aligners limits opportunities for reuse or recycling, resulting in this nonbiodegradable waste ending up in landfills, oceans, and incineration pathways [6–8]. Over time, their long‐term environmental persistence contributes to plastic pollution in terrestrial and aquatic environments, with potential implications for ecological systems, including marine life [6]. Beyond this, the environmental impact of clear aligner systems extends across the broader product life cycle, including raw material extraction, manufacturing, transportation, and distribution [2, 3, 9]. The reliance on petroleum‐based polymers, energy‐intensive production processes, and global supply chains contributes to greenhouse gas emissions and resource depletion. These concerns position CAT within the wider discourse on sustainable healthcare, where high‐volume, disposable medical products are increasingly scrutinized for their ecological impact [2, 5].

Moreover, aligners contribute to the release of microplastics and nanoplastics, both within the oral cavity due to mechanical wear during treatment and through broader environmental contamination [3, 7, 10]. The degradation of plastic materials used in aligners may result in the release of microplastics into the oral cavity and within the external environment, posing a risk of ingestion and systemic exposure and potentially leading to long‐term health effects [3]. Furthermore, concerns have been raised regarding the leaching of chemical substances such as bisphenol A (BPA) and other endocrine‐disrupting compounds from polymers [11].

With the rapid expansion of CAT and the emerging concerns related to its environmental and health impacts, the existing literature remains fragmented, where current research is dispersed across multiple areas, including in vitro studies, materials science, environmental impact, and regulatory discourse, with limited integration of these perspectives to provide a comprehensive understanding of the broader impacts of CAT. Accordingly, this review seeks to consolidate and integrate evidence across clinical, material, environmental, and regulatory domains to provide a comprehensive and cohesive understanding of the broader implications of CAT. In this context, this scoping review aimed to systematically map and synthesize the available evidence addressing the research question: “What is currently known about the environmental and public health implications of CAT across clinical, manufacturing, environmental, and regulatory contexts?” By identifying key themes, knowledge gaps, and areas of emerging concern, this review aimed to inform future research, guide policy and regulatory frameworks, and support the development of more sustainable and responsible orthodontic practices.

## 2. Materials and Methods

This scoping review followed the Arksey and O’Malley framework [12] for conducting scoping reviews and was reported in line with PRISMA‐ScR guidelines [13]. A protocol was developed a priori to guide the review; however, it was not formally registered.

### 2.1. Identifying the Research Question

In line with the first stage of the Arksey and O’Malley framework, the following research question was identified for this scoping review: “What is currently known about the environmental and public health implications of CAT across clinical, manufacturing, environmental, and regulatory contexts?”

### 2.2. Eligibility Criteria

This research question, which aimed to broadly map the existing literature, was further structured utilizing the Population‐Concept‐Context (PCC) framework recommended by the Joanna Briggs Institute for scoping reviews [14]. The PCC framework was used to facilitate a clearer understanding of the eligibility criteria to be applied during the screening of the literature for this review. Based on this framework, the eligibility criteria are presented below.

Population: the review included literature involving one or more of the following populations or stakeholders:

1. Patients undergoing orthodontic treatment using clear aligners.
2. Dental and orthodontic professionals.
3. Manufacturers and suppliers of clear aligner systems.
4. Environmental, public health, and regulatory stakeholders relevant to dental materials and waste.

Concept: studies were included if they addressed at least one of the following concepts related to CAT:

1. Environmental impact.
2. Plastic waste generation and disposal.
3. Microplastic release or exposure.
4. Chemical leaching (e.g., BPA and related compounds).
5. The public health risks or implications.
6. Sustainability and environmental responsibility.
7. Policy, regulatory, or governance considerations.

Context: all contextual settings were considered eligible for inclusion in the scoping review, including the following:

1. Clinical and dental practice settings.
2. Manufacturing and material production environments.
3. Environmental and ecological contexts.
4. Regulatory, public health, and policy frameworks.

This scoping review included a broad range of study types to comprehensively capture the existing literature relevant to the aligner materials, environmental sustainability, or associated public health concerns of CAT. These sources included original research studies such as laboratory, observational, and experimental designs, as well as all types of review articles such as narrative, systematic, and umbrella reviews. Environmental assessments, policy analysis, regulatory reports, opinion pieces, editorials, letters to the editor, short communications, or commentaries were also considered for inclusion if they contained relevant conceptual, contextual, or practice‐related information that informed the domains of the review. Articles published in English, either in print or published online ahead of print, between January 2005 and December 2025, were included in this scoping review. The past two decades were considered as they roughly correspond with the rapid growth of CAT and increasing attention to sustainability and environmental considerations within healthcare.

Studies focused on nondental plastics, orthodontic appliances other than clear aligners, or exclusively on attachments used during CAT were excluded from the review. Publications in languages other than English and studies for which the full text was either unavailable or inaccessible were also excluded from this scoping review. Additionally, publication types, such as product news, which did not contribute meaningful evidence or contextual insight to address the research question, were excluded.

### 2.3. Information Sources and Search Strategy

A comprehensive literature search was conducted across four electronic databases by one author (Sakshi Sakshi) to identify the relevant studies, namely, PubMed, Embase, Scopus, and Web of Science, up to December 2025. Search strategies were designed to ensure comprehensive coverage of relevant concepts utilizing a combination of Medical Subject Headings (MeSH) and synonyms of free‐text keywords combined using Boolean operators. Key search terms included “orthodontic appliances, removable” [MeSH] OR “clear aligners” OR “Invisalign” OR “orthodontic aligner” OR “clear aligner therapy” along with varying combinations of terms such as “microplastics” [MeSH], “recycling” [MeSH], “environmental impact,” “waste disposal,” “dental waste,” “waste management,” “sustainability,” “bisphenol A,” “chemical leaching,” “toxicity,” “public health,” “policy,” “health policy,” and “health care policy,” with appropriate Boolean operators applied across the search. The details of the search strategy across the four databases are provided in Supporting Information 1: File S1. In addition, a gray literature search and manual reference screening were not undertaken for this review, and no major methodological deviations occurred during the review process.

### 2.4. Study Selection

All the search results were imported into Rayyan software for the screening process [15]. Rayyan automatically detects duplicate records, which were resolved manually by the authors where required. Following the removal of duplicates, title and abstract screening was performed independently by two reviewers (Sakshi Sakshi and Asavari Desai) to assess the articles for inclusion into the scoping review based on the predefined eligibility criteria. Articles selected at this stage underwent full‐text screening, which was also conducted independently by the two reviewers (Sakshi Sakshi and Asavari Desai) to determine final inclusion into the scoping review for data synthesis. Any disagreements at either stage of screening were resolved through discussion, and where consensus could not be reached, a third reviewer (Parul Dasson Bajaj) was consulted.

### 2.5. Charting the Data

Data extraction was conducted by two independent reviewers (Sakshi Sakshi and Parul Dasson Bajaj) utilizing a predecided data charting form. The following variables were extracted from each included study: author(s) and year of publication, country or geographic region, study design or type of article, aims and objectives, methodology, type of clear aligner material or system examined, environmental impact indicators (e.g., waste generation, microplastic release, chemical leaching, and carbon footprint where reported), public health/policy/regulatory/governance issues addressed, key findings, limitations, or research gaps identified. The extracted data were subsequently compared to ensure consistency and accuracy. Any discrepancies in data extraction were resolved through discussion and mutual agreement between the two reviewers.

### 2.6. Synthesis of Results

The included studies were synthesized using a qualitative thematic analysis approach [16] to identify and map recurring patterns and key concepts, which addressed the research question and the conceptual scope of the review. Following data charting, the extracted information along with the full‐text articles was analyzed iteratively by one researcher (Parul Dasson Bajaj) using ATLAS.ti 25.0.2 for Mac [17]. The initial coding was conducted by one researcher (Parul Dasson Bajaj) to identify the initial codes, which were subsequently revisited to arrive at the final codes. At this stage, two additional researchers (Sakshi Sakshi and Asavari Desai) were involved to refine the coding and group these codes into broader themes reflecting the major areas of discussion within the evidence base.

## 3. Results

### 3.1. Study Selection Process

**Figure 1:**
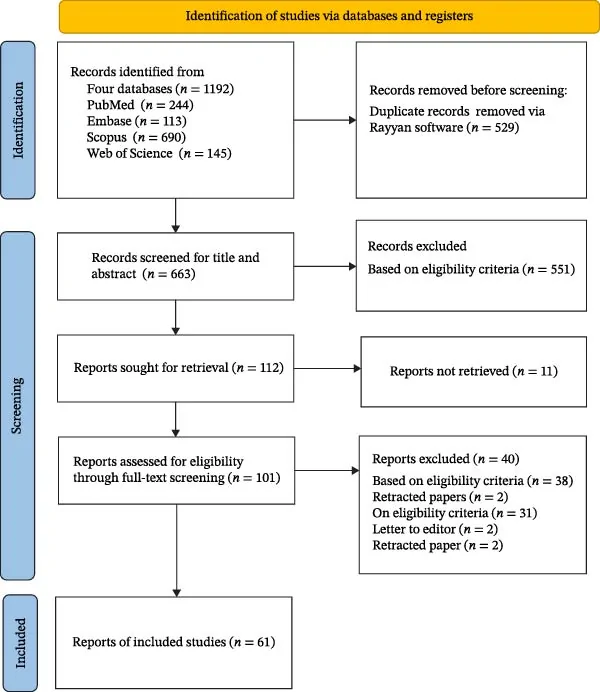
PRISMA flow diagram illustrating the study selection process.

The initial search yielded a total of 1192 articles. Following duplicate removal using Rayyan software, 663 records remained and were subjected to title and abstract screening. Of these, 112 records were selected for full‐text retrieval; however, 11 records could not be accessed or retrieved. As a result, 101 records underwent full‐text screening based on the eligibility criteria, of which 61 records were finally included in the scoping review. The study selection process is illustrated in the PRISMA flow diagram (Figure 1) [18].

### 3.2. Characteristics of Sources of Evidence

A total of 61 studies published between 2009 and 2026 were included in this scoping review. The temporal distribution of the included literature indicates that this area of research is relatively recent and rapidly expanding. Among the selected evidence, only one paper was published in 2009 [19], followed by two studies in 2019 [20, 21] and one study in 2020 [22]. Publication output increased notably from 2022 onward, with 8 articles published in 2022 [8, 23–29], 9 in 2023 [6, 10, 30–36], and 11 in 2024 [2, 7, 37–45], before rising sharply to 27 studies in 2025 [3, 5, 11, 46–69] and 2 studies in 2026 [70, 71]. This sharp upward trend highlights the growing recognition of the environmental, material‐related, and public health concerns associated with CAT. The details of the characteristics of the sources of evidence, along with the extracted data, are presented in Supporting Information 2: File S2.

The included evidence predominantly consisted of in vitro studies (*n* = 27), reflecting a strong focus on laboratory‐based evaluation of cytotoxicity, chemical leaching, material degradation, and microplastic release [10, 19, 20, 23, 24, 28, 29, 33, 35, 39, 41, 42, 44, 46–48, 52, 56, 59, 61–64, 68–71]. A considerable proportion of the literature consisted evidence synthesis with review‐based papers including 9 systematic reviews [25, 34, 36, 49, 50, 53, 57, 58, 65], 1 systematic review and meta‐analysis [22], 1 scoping review [3], 2 umbrella reviews [11, 60], 11 narrative reviews [2, 5–7, 27, 31, 32, 37, 51, 55, 66], and 1 critical review [54]. The remaining studies included three editorials [26, 40, 45], one letter to the editor [43], one short communication [8], one life cycle assessment [30], one technical development [21], one ex vivo study [38], and one prospective observational study [67]. Collectively, this distribution indicates that the field is currently driven largely by laboratory‐ and review‐based evidence, while clinical, translational, life cycle, and policy‐focused research remains comparatively limited.

The included studies examined a wide range of aligner materials, predominantly comprising thermoformed polymers such as PET and PET‐G [6, 20, 22, 30, 31, 34, 36, 38, 41, 44, 47, 50–53, 57, 61, 62, 65–67, 69], polyurethane‐based systems including TPU [6, 24, 31, 33, 34, 41, 46, 50, 52, 53, 59–63, 65–67], and multilayer polymers [39, 46, 57, 59, 65–68]. Emerging materials included directly 3D‐printed photopolymer resins [21, 25, 29, 30, 35, 37, 42, 48, 49, 54, 57, 58, 64, 65, 68, 70, 71], which represent a rapidly growing area of research, as well as shape‐memory and 4D aligner systems [42, 51, 65]. In addition, several studies evaluated alternative or modified materials, including BPA‐free resins and nanoparticle‐coated polymers designed to enhance biocompatibility and functionality [21, 64]. A significant proportion of studies assessed commercial aligner systems where material composition is not fully disclosed, thereby limiting direct comparison and standardization across the evidence base.

### 3.3. Qualitative Thematic Synthesis of the Included Studies

The thematic framework (Table 1), emerging from the qualitative thematic analysis of the sources of evidence, reveals a multilevel evidence structure regarding CAT with interrelated main themes, namely, (1) material toxicity and chemical exposure, (2) microplastic and nanoplastic release, (3) environmental burden of CAT, (4) technological transitions in the form of 3D/4D aligners, (5) strategies toward sustainability, and (6) governance, regulation, and research gaps.

**Table 1:** Overview of thematic structure from qualitative thematic analysis.

Main themes	Subthemes	References
Material toxicity and chemical exposure	Chemical leaching from polymers	[11, 22, 24–26, 33–39, 49, 52, 53, 57, 60, 63, 67, 71]
	Cytotoxicity and estrogenicity	[6, 11, 19, 20, 23, 25, 28, 29, 32, 35, 37, 41, 44, 46, 48, 49, 52–54, 57–61, 70]
	Exposure conditions and cumulative risk	[22, 24, 36, 38, 45, 49, 53, 60, 67]
Microplastic and nanoplastic release	Mechanical wear	[3, 10, 40, 47, 50, 66, 68]
	Biological and systemic implications	[6, 7, 10, 40, 47, 50, 66, 69]
	Manufacturing and material variability	[10, 68]
Environmental burden of clear aligner therapy	Plastic waste generation	[2, 3, 5–8, 26, 27, 31, 32, 37, 51]
	Life‐cycle impact	[30]
	Waste disposal and emissions	[3, 6, 7, 26, 27, 31, 32, 62]
Technological transitions (3D/4D aligners)	Benefits of new technologies	[2, 3, 5, 6, 21, 30, 37, 42, 62, 65, 69]
	Risks and contradictions	[3, 6, 25, 35, 49, 51, 52, 54, 57, 58, 65, 71]
	Process dependency	[3, 6, 35, 49, 54, 57, 58]
Strategies toward sustainability	Recycling and waste management	[2, 3, 5, 6, 8, 26, 27, 32, 37, 43, 45, 55, 56, 62]
	Material innovation	[2, 3, 5, 6, 37, 51, 55, 62, 65]
	Clinical strategies	[2, 3, 5, 7, 8, 37, 62, 71]
Governance, regulation, and research gaps	Regulatory and policy challenges	[3, 8, 26, 27, 43, 52, 54, 56, 62, 68]
	Material transparency issues	[30, 34, 44, 46, 49, 51, 52, 54, 57, 59]
	Research and methodological gaps	[6, 8, 11, 19, 20, 22–25, 28, 29, 34, 36–38, 41, 46, 49, 50, 53, 54, 57, 58, 60, 63, 66–71]

The first main theme, at the foundational level, relates to the material composition and processing of aligners, which influences chemical leaching and cytotoxicity of aligner materials. Clear aligners have been associated with leaching of toxic chemicals often used as additives and residual monomers, such as BPA, isocyanates, urethane di‐methacrylate (UDMA), and related substances. The studies included in this scoping review reveal that the extent of this release is influenced by intraoral conditions, time‐dependent degradation patterns, and environmental factors, such as pH, temperature, and solvent exposure. The potential biological effects of these leached compounds are reflected in studies evaluating cytotoxicity and estrogenicity, which demonstrate heterogeneous findings. Overall, these findings suggest that these effects are material‐specific and concentration‐dependent effects, and toxicity may evolve over time with repeated aligner replacement, as seen in CAT every 7–14 days.

The second main theme encompasses microplastic and nanoplastic release, focusing on the mechanical wear of aligners within the oral cavity during routine use under varying intraoral conditions. These particles introduce potential pathways for ingestion, systemic exposure, and biological interaction, including effects on the gut microbiota and inflammatory processes, although direct clinical evidence remains limited. The biological impact of these particles is further influenced by their size, with smaller particles potentially exhibiting a greater capacity for systemic interaction. Additionally, differences in the method of manufacturing appear to influence particle release, with directly printed aligners releasing more microplastics in comparison to thermoformed aligners, likely due to variations in surface characteristics.

Moving beyond health implications, the third main theme considers the environmental burden posed by CAT due to the high‐volume, nonbiodegradable, polymer‐intensive system with significant waste generation. The waste is not only generated from used aligners but also from thermoforming processes and 3D model fabrication. The environmental burden extends beyond waste disposal to encompass life cycle impacts such as energy use, carbon emissions, and resource depletion. Furthermore, improper waste disposal and incineration were associated with secondary environmental hazards, including the release of toxic compounds such as benzene, cyanides, and tetrahydrofuran.

The fourth main theme involves technological innovation in aligner materials, particularly the transition toward direct 3D printing and emerging 4D/shape‐memory aligners, which represent an effort toward reducing the environmental burden and moving toward sustainability. While these approaches may reduce material waste and improve manufacturing efficiency, they also introduce new safety concerns related to residual monomers, incomplete polymerization, and process‐dependent toxicity. This highlights the contradictions within the evidence base, with innovations aimed at improving sustainability that might simultaneously generate new forms of public health risk.

The fifth main theme focuses on strategies toward sustainability, including recycling initiatives, chemical and biological degradation methods, and material innovations. In addition, this theme highlights system‐level optimization, incorporating clinical strategies involving clinical decision‐making, treatment planning, patient compliance, and production models. Finally, the sixth theme, governance, regulation, and research gaps, which collectively inform future directions in this field. The current evidence base is characterized by limited transparency regarding material composition, a predominance of in vitro studies, insufficient clinical validation, and a lack of standardized testing protocols.

In Section 4, these themes and key findings would be examined within the context of global literature together using an integrated approach, recognizing that these themes are inherently interconnected and collectively shape the sustainability and public health implications of CAT.

## 4. Discussion

This scoping review provides an overview of the current evidence base on the environmental and public health implications of CAT, suggesting that its relevance may extend beyond its role as a clinical orthodontic intervention to include broader material, environmental, and public health implications. The findings highlight a multilayered evidence structure spanning interdependent themes, including material composition and toxicity, microplastic release, environmental burden, technological innovation, strategies toward sustainability, and governance and regulatory gaps in aligner disposal as well as broader research gaps. Accordingly, this section examines these themes in an integrated manner, exploring how they interact and contribute to a comprehensive understanding of CAT.

This review mapped the available evidence across four predefined domains: clinical, manufacturing, environmental, and regulatory contexts. The clinical domain considered the potential implications of CAT materials for patient care, although direct clinical evidence was limited, with only two in vivo studies identified [38, 67]. Practice‐related considerations were therefore informed largely by the findings from studies examining material characteristics, manufacturing processes, and associated biological and environmental effects. The manufacturing domain, which comprised the largest proportion of the available evidence, focused on the materials, fabrication methods, and production processes used in aligner development, predominantly through in vitro investigations. The environmental domain included evidence relating to plastic waste, microplastic release, recycling, and emerging approaches intended to reduce the environmental burden of CAT while also reflecting the influence of both manufacturing decisions and patterns of clinical use. However, it was inseparable from both the manufacturing domain as well as clinical usage. The regulatory domain encompassed standards and guidance relevant to material safety, biocompatibility, manufacturing, marketing, and clinical use. However, regulatory guidance concerning the collection, recycling, and disposal of used aligners is limited, inconsistently documented, and characterized by substantial gaps in end‐of‐life management. Taken together, these findings illustrate that the four domains, although presented separately for analytical clarity, are closely interconnected and are best understood as interdependent components of the broader CAT lifecycle.

The broader perspective emerging from this scoping review aligns with the World Health Organization’s Global Oral Health Action Plan 2023–2030, which advocates for sustainability in oral healthcare services to minimize environmental impact [72]. However, in the context of CAT, sustainability cannot be considered in isolation and must be examined as a multidimensional issue shaped by the interplay between material composition and innovation, clinical considerations, manufacturing processes, public health implications, and the policy or regulatory frameworks that govern them, as discussed above.

As highlighted by the evidence in this scoping review, a meaningful transition toward sustainability in CAT will require the coordinated involvement of all relevant stakeholders and cannot be achieved through isolated action at a single level [3, 6, 37, 45]. Therefore, the following sections examine sustainability across the full stakeholder continuum, from patients and clinicians to manufacturers, researchers, policymakers, and regulators, considering the opportunities, challenges, and interdependencies at each level within the broader thematic structure of the review findings.

Clear aligners enter the care pathway at the stage where clinicians and patients choose CAT as a treatment modality. At this stage, clinicians could responsibly make appropriate choices in case selection and treatment planning, taking into account not only expected treatment outcomes but also the available evidence relating to the potential biological implications of plastic and chemical leaching from aligner materials within the oral cavity as well as the potential environmental burden associated with aligner use [62]. These considerations may be particularly relevant given that treatment with CAT is primarily sought for esthetic reasons, despite the availability of well‐established alternative treatment modalities [7, 8]. Careful case selection is especially important in situations where aligner therapy is anticipated to require extended treatment times in comparison with fixed appliances [2]. Importantly, wherever deemed appropriate, this informed decision‐making could extend beyond clinicians to include patients, who may be educated about the health and environmental implications of CAT and provided with a balanced understanding of the available treatment options. Such shared decision‐making would ensure that choices are made with due consideration for patient safety, clinical efficacy, and broader sustainability concerns [7].

Beyond the choice of treatment, clinicians can adopt additional strategies to reduce waste during CAT. One such approach is the staged production of aligners rather than ordering the entire set at once, which can help in managing tracking failure while minimizing waste from unused aligners [3, 4]. This approach provides greater flexibility, allowing orthodontists to modify treatment plans in response to various factors like case complexity, incorrect impressions, patient noncompliance, lost aligners, discrepancies between digital simulation and actual tooth movement, additional dental procedures, or the need for refinements, while simultaneously avoiding unnecessary waste from unused aligners, including the associated manufacturing and packaging burden [2, 5, 73]. Evidence also suggests that efforts should be made to reduce the number of aligners along with the overall treatment duration. However, achieving this will depend partly on research and development by the manufacturers, who should prioritize innovations that reduce the number of aligners and improve treatment efficiency rather than increasing aligner frequency [8, 37, 71]. Patients also play a key role as compliance is critical to successful aligner therapy; therefore, improving compliance enhances both treatment efficiency and sustainability. Inadequate aligner wear that is less than 20 h per day can lead to tracking failure, necessitating additional aligners and thereby increasing the waste from both unused and additionally manufactured aligners [2, 3].

Patient education should extend beyond increasing compliance to include the material properties and safe handling of aligners. This is of particular importance as thermoplastic aligners may degrade under intraoral conditions such as high temperature and acidic environments, and patients should therefore be advised accordingly [25, 53]. Such guidance becomes relevant in light of concerns regarding the release of cytotoxic monomers, microplastics, and other material by‐products, which highlights the need for patients to understand how aligners should be used and maintained. This aligns with the evidence included in this scoping review, which suggests that the aligner materials evaluated across studies ranged from no cytotoxic potential [2, 19, 37, 44, 61] to slight cytotoxicity [6, 20, 23, 41, 46, 53, 57, 59]. However, most of these findings were derived from in vitro studies and did not take into account the dynamic intraoral environment, which remains an important limitation of the current evidence base [11, 32, 54, 71].

Patient education should be further supplemented by information regarding the potential health implications of chemical and microplastic release from aligner materials, as well as the broader environmental consequences of aligner use, including the need for responsible disposal of used aligners. In this regard, manufacturers could contribute by placing mandatory return or disposal warnings on aligner packaging to encourage more sustainable waste management after use [5, 62]. Manufacturers and the companies supplying clear aligners may also limit the number of aligners provided to clinicians rather than allow unrestricted access under the current practice [5, 6]. They can also provide guidance to clinicians regarding the safe disposal and recycling of used aligners, which can be in turn communicated to patients [6]. Given that used aligners may be classified as medical waste, clinicians can further support safer disposal by patient education and providing guidance on cleaning and disinfection before recycling, as described by one such paper included in this review, which elaborates on a standard operating procedure for managing aligner waste prior to recycling [27]. Additionally, clinicians can support sustainability by establishing collection units for used aligners within their practices [5, 37, 62]. Examples of such sustainability initiatives have been reported in the literature, including free recycling programs through partnerships between orthodontic practices, aligner manufacturers, and companies such as TerraCycle [3, 5, 74, 75].

While disposal practices are important, sustainability in CAT may also be influenced by upstream decisions relating to the materials and manufacturing systems used to produce aligners. In CAT, the predominantly used aligners are thermoformed, and their manufacturing process generates a considerable amount of waste. This waste arises not only through the aligners themselves but also from excess plastic discarded from thermoforming sheets and from 3D‐printed models used during fabrication [5]. These concerns have prompted interest in more circular approaches, such as the use of recycled materials for the fabrication of 3D models required in aligner production [2, 6, 37]. Directly printed aligners or 3D‐printed aligners have also been proposed as a potential means of reducing some of the material and process requirements of thermoforming, like the need for 3D models. This may thereby help in reducing costs, waste generation, and carbon footprint by lowering water, raw material, and energy consumption [2, 3, 5, 6, 30, 62, 65].

However, the potential environmental advantages of directly printed aligners need to be considered alongside their material and biological characteristics. The available evidence is derived predominantly from in vitro studies and remains insufficient to support definitive conclusions regarding cytotoxicity. Nevertheless, some studies have reported differences in biological responses between directly printed and thermoformed aligners, with certain directly printed materials demonstrating comparatively greater cytotoxic effects under the conditions investigated [6, 25, 29, 35, 48, 54, 58, 70]. In addition, residual uncured resins may remain on the surfaces of 3D‐printed aligners, highlighting the importance of following postcuring processes recommended by the manufacturers as essential for enhancing their safety profile [49, 54, 58]. Variability in mechanical properties due to printer‐specific differences represents another important limitation of these materials [3]. Evidence from an in vitro study has suggested greater microplastic release from directly printed aligners than from thermoformed alternatives [68]. Although reports in the literature, not concerning aligners per se but the ingestion of microplastics, have raised concerns regarding the possible systemic effects of microplastic exposure, including effects on the gastrointestinal, respiratory, cardiovascular, and neurological systems, the clinical significance of exposure arising specifically from aligner use remains uncertain [7].

A further challenge at the manufacturing level relates to the limited transparency around aligner material composition. Manufacturers often do not fully disclose proprietary information regarding the chemical composition of the aligner materials, which poses a significant barrier to both research and sustainability efforts [52, 54]. This lack of transparency restricts direct comparison across materials, complicates the evaluation of cytotoxicity and other biological effects, and creates additional challenges for recycling as different materials may generate distinct by‐products and may require different recycling pathways [44, 54, 55, 59, 62].

Additionally, sustainability in CAT would also require research and development toward more efficient, eco‐friendly, and biocompatible materials [62]. One such emerging innovation is the development of shape‐memory polymers or 4D‐printed aligners, which may help reduce waste by enabling the use of a single shape‐changing aligner in place of three conventional aligners while also eliminating the need for 3D‐printed dental models for production [2, 3, 5, 6, 42, 51, 76] However, like directly printed aligners, these materials also introduce their own set of issues, including incomplete polymerization, release of residual monomer, printer‐specific inconsistencies, shape‐memory fatigue, and inconsistent force delivery, all of which may affect the mechanical performance and clinical safety of these aligners [3].

Alongside these developments, a range of eco‐friendly and biocompatible materials are being explored, such as silk fibroin and soybean‐based polymers, as well as monomer‐free biocompatible 3D‐printed resins, with an aim to address both sustainability and toxicity concerns. These materials have been proposed as possible approaches to addressing concerns related to both environmental sustainability and biological compatibility [2, 3, 6, 77, 78]. Although such biodegradable and bio‐based plastics may help reduce environmental impact, microplastic release due to intraoral friction may still be a concern and warrant further investigation [2]. Additionally, during the development of newer materials, there is a need to maintain key mechanical properties, including strength, transparency, force delivery, and dimensional stability, without compromising treatment efficiency, thereby necessitating further evaluation of the feasibility of orthodontic treatment and long‐term performance of these materials [5, 78]. Furthermore, the recycling of these newer materials may increase overall cost as it might require specialized processing and infrastructure [9].

With regard to recycling practices, conventional mechanical recycling of small plastics such as aligners and their packaging is often less favorable, as the energy required for recycling frequently may exceed the material recovered [4]. This has led to increasing interest in alternative recycling and degradation methods, such as alkaline hydrolysis and microbial degradation of used plastics [47, 55, 62, 79]. Evidence from this scoping review suggests that such methods may offer safer and potentially sustainable pathways for reducing the amount of aligner waste, which ends up in landfills [47, 55]. This is particularly important because used aligners, which may be regarded as medical waste, frequently enter regular waste streams due to improper disposal, where they are either sent to landfill or incinerated, leading to the release of harmful compounds such as benzene and tetrahydrofuran, both of which are recognized carcinogens and are also associated with other adverse biological effects [62].

Overall, the evidence from this scoping review draws attention to the need for a broader systems‐level response, in which sustainability in CAT is supported by stronger governance, clearer regulation, and decisive policymaking. Without stringent regulation, the growing use of nonbiodegradable aligners will continue to add to global plastic waste and may amplify the associated health risks [5, 62]. Policymakers therefore play a critical role in enforcing appropriate medical waste disposal requirements and in supporting the green innovations and recycling infrastructure needed for sustainable aligner use [3, 31]. The findings of this review suggest that guidance concerning the classification, collection, and disposal of used aligners remains limited and inconsistently addressed. Clearer regulatory provisions may therefore be needed to determine whether, and under what circumstances, aligners should be managed as controlled medical waste to reduce the likelihood of inappropriate disposal into the environment [43]. Policies and regulatory frameworks must also move beyond disposal alone to require greater manufacturer accountability and transparency while establishing standardized safeguards for material toxicity, long‐term chemical leaching, and microplastic exposure [3, 8, 53, 66]. These efforts would benefit from additional clinical research, including well‐designed prospective studies and, where feasible, randomized controlled trials, given that the current evidence is derived predominantly from in vitro investigations. Ultimately, protecting public health and ensuring environmental sustainability will require a coordinated approach that brings together scientific research, industry responsibility, and firm regulatory oversight.

The evidence from this review highlights the need for future research to move beyond short‐term in vitro studies toward robust in vivo and clinical studies, particularly randomized controlled trials, to more accurately examine the biocompatibility and systemic effects of clear aligners [22, 25, 38, 60, 71]. There is a need to develop standardized testing protocols to simulate the dynamic oral environment, which is essential for evaluating chemical leaching and microplastic release [57, 66]. A major gap also remains in understanding the clinical release and biological significance of microplastics and nanoplastics, particularly their systemic exposure and accumulation over time [57, 66]. Emerging alternative materials such as directly printed and 4D shape‐memory aligners require rigorous long‐term evaluation of their mechanical performance, clinical effectiveness, and biological safety, including optimization of postcuring procedures to minimize residual monomer release [37, 54, 57, 65]. Additionally, sustainability research should prioritize comprehensive life cycle assessments and scalable waste management solutions, including microbial degradation, alkaline hydrolysis, and development of clinically viable biodegradable materials [3, 55, 56].

While the findings of this review provide an important synthesis of the available evidence, this scoping review had several limitations, which should be considered. First, the search was restricted to four databases and publications in the English language. In addition, gray literature was not searched, and manual reference screening was not undertaken, which may have limited the identification of additional relevant sources. In addition, although an a priori protocol was developed to guide the review process, it was not formally registered. Nevertheless, no major methodological deviations occurred during the conduct of the review. Second, the available evidence was largely dominated by in vitro studies, with relatively few in vivo or clinically relevant investigations, which constrains the extent to which laboratory findings can be extrapolated to real‐world intraoral conditions. This limitation is further compounded by substantial heterogeneity across experimental protocols, materials studied, and generally short study durations, which hinder comparisons across studies and limit the ability to draw definitive conclusions. In addition, the review included diverse publication types, such as narrative reviews, editorials, letters, and short communications. Although their inclusion was appropriate for a scoping review intended to map the breadth of the literature, no formal quality appraisal was performed as this is not a mandatory step in the scoping review methodology. Collectively, these limitations indicate that the current evidence base remains preliminary. The findings of this review should therefore be interpreted with caution. Future research should prioritize standardized and reproducible in vitro protocols and well‐designed long‐term clinical studies under real‐world intraoral conditions to assess cumulative effects over time, along with life cycle and regulatory assessments across different geographic settings. Such research would strengthen the evidence base and support more informed clinical, environmental, and policy decision‐making.

## 5. Conclusions

CAT has emerged as a widely accepted orthodontic modality, yet this review suggests that its environmental, public health, and regulatory implications have not kept pace with its clinical and commercial expansion. The evidence mapped in this review points to growing concerns around intensive production and use of nonbiodegradable plastics, waste generation, chemical leaching, and the possible release of micro‐ and nanoplastics, while also revealing significant gaps in long‐term clinical evidence, environmental monitoring, and policy oversight. Collectively, these findings suggest that clear aligners should no longer be viewed solely through the lens of esthetics and treatment efficiency but also as part of a broader sustainability and health discourse, with important implications for Sustainable Development Goal 3 concerning good health and well‐being and Sustainable Development Goal 13 on climate action. Addressing these challenges will require coordinated action across research, clinical practice, manufacturing, and governance to ensure that future dental innovations are not only effective and patient‐centered but also biocompatible, environmentally responsible, and aligned with the evolving global public health and environmental sustainability priorities.

## Author Contributions

Sakshi Sakshi contributed to conceptualization, methodology, investigation, formal analysis, and writing – original draft. Asavari Desai contributed to conceptualization and design, methodology, formal analysis, writing – review and editing, and supervision. Parul Dasson Bajaj contributed to investigation, formal analysis, and writing – review and editing.

## Funding

This review did not receive any funding support.

## Disclosure

All authors read and approved the final version of the manuscript. An earlier version of this article was presented at the IAPHD NATCON 2025 (Conference ID 600).

## Conflicts of Interest

The authors declare no conflicts of interest.

## Supporting Information

Additional supporting information can be found online in the Supporting Information section.

## Supporting information


**Supporting Information 1** File S1: This file contains the details of the search strategy across the four databases.

**Supporting Information 2** File S2: This file presents the details of the characteristics of the sources of evidence and the data extracted from each source.

## Data Availability

Data are available in the article supporting information.
