# Supplementary material for: The Environmental Burden and Sustainability Challenges of Clear Aligners: Implications for Public Health, Practice, Policy, and Future Research—A Scoping Review
Source: Int J Dent. 2026 Aug 30;2026:2681058. doi: 10.1155/ijod/2681058 (PMC13526242; doi:10.1155/ijod/2681058)
Supplement: Supplementary file 1 [file IJOD-2026-2681058-s001.docx]

**Search String Across Various Databases**

| **Database** | **Fields Chosen** | **Date** | **Search String** | **Search Results** |
| --- | --- | --- | --- | --- |
| PubMeD | All Fields | December 2025 | (((((orthodontic aligner) OR (clear aligner)) OR (clear aligner appliance)) OR (clear aligner therapy)) OR (invisalign)) AND (microplastics) | 11 |
|  |  |  | (((((orthodontic aligner) OR (clear aligner)) OR (clear aligner appliance)) OR (clear aligner therapy)) OR (invisalign)) AND (bisphenol a) | 27 |
|  |  |  | (((((orthodontic aligner) OR (clear aligner)) OR (clear aligner appliance)) OR (clear aligner therapy)) OR (invisalign)) AND (toxicity) | 114 |
|  |  |  | (((((orthodontic aligner) OR (clear aligner)) OR (clear aligner appliance)) OR (clear aligner therapy)) OR (invisalign)) AND (sustainable orthodontics) | 44 |
|  |  |  | (((((orthodontic aligner) OR (clear aligner)) OR (clear aligner appliance)) OR (clear aligner therapy)) OR (invisalign)) AND (recycling) | 23 |
|  |  |  | (((((orthodontic aligner) OR (clear aligner)) OR (clear aligner appliance)) OR (clear aligner therapy)) OR (invisalign)) AND (waste management) | 25 |
| Embase | All Fields | December 2025 | ('orthodontic aligner'/exp OR 'orthodontic aligner' OR 'clear aligner therapy'/exp OR 'clear aligner therapy' OR 'invisalign'/exp OR 'invisalign') AND ('microplastic'/exp OR 'microplastic') | 9 |
|  |  |  | ('orthodontic aligner'/exp OR 'orthodontic aligner' OR 'clear aligner therapy'/exp OR 'clear aligner therapy' OR 'invisalign'/exp OR 'invisalign') AND ('microplastic pollution'/exp OR 'microplastic pollution') | 2 |
|  |  |  | ('orthodontic aligner'/exp OR 'orthodontic aligner' OR 'clear aligner therapy'/exp OR 'clear aligner therapy' OR 'invisalign'/exp OR 'invisalign') AND ('environmental impact'/exp OR 'environmental impact') | 4 |
|  |  |  | ('orthodontic aligner'/exp OR 'orthodontic aligner' OR 'clear aligner therapy'/exp OR 'clear aligner therapy' OR 'invisalign'/exp OR 'invisalign') AND ('public health'/exp OR 'public health') | 56 |
|  |  |  | ('orthodontic aligner'/exp OR 'orthodontic aligner' OR 'clear aligner therapy'/exp OR 'clear aligner therapy' OR 'invisalign'/exp OR 'invisalign') AND ('bisphenol a'/exp OR 'bisphenol a') | 12 |
|  |  |  | ('orthodontic aligner'/exp OR 'orthodontic aligner' OR 'clear aligner therapy'/exp OR 'clear aligner therapy' OR 'invisalign'/exp OR 'invisalign') AND ('toxicity'/exp OR 'toxicity') | 18 |
|  |  |  | ('orthodontic aligner'/exp OR 'orthodontic aligner' OR 'clear aligner therapy'/exp OR 'clear aligner therapy' OR 'invisalign'/exp OR 'invisalign') AND ('sustainability'/exp OR 'sustainability') | 3 |
|  |  |  | ('orthodontic aligner'/exp OR 'orthodontic aligner' OR 'clear aligner therapy'/exp OR 'clear aligner therapy' OR 'invisalign'/exp OR 'invisalign') AND ('environmental sustainability'/exp OR 'environmental sustainability') | 1 |
|  |  |  | ('orthodontic aligner'/exp OR 'orthodontic aligner' OR 'clear aligner therapy'/exp OR 'clear aligner therapy' OR 'invisalign'/exp OR 'invisalign') AND ('recycling'/exp OR 'recycling') | 3 |
|  |  |  | ('orthodontic aligner'/exp OR 'orthodontic aligner' OR 'clear aligner therapy'/exp OR 'clear aligner therapy' OR 'invisalign'/exp OR 'invisalign') AND ('waste management'/exp OR 'waste management') | 4 |
|  |  |  | ('orthodontic aligner'/exp OR 'orthodontic aligner' OR 'clear aligner therapy'/exp OR 'clear aligner therapy' OR 'invisalign'/exp OR 'invisalign') AND ('waste disposal'/exp OR 'waste disposal') | 1 |
| Scopus | All Fields | December 2025 | orthodontic appliances , removable OR orthodontic appliance design OR clear aligner OR invisalign AND microplastics | 29 |
|  |  |  | orthodontic appliances , removable OR orthodontic appliance design OR clear aligner OR invisalign AND environment AND public health OR environmental impact | 129 |
|  |  |  | orthodontic appliances , removable OR orthodontic appliance design OR clear aligner OR invisalign AND microplastics AND environment AND public health OR environmental impact | 18 |
|  |  |  | orthodontic appliances , removable OR orthodontic appliance design OR clear aligner OR invisalign AND marine environment | 17 |
|  |  |  | orthodontic appliances , removable OR orthodontic appliance design OR clear aligner OR invisalign AND terrestrial environment | 5 |
|  |  |  | orthodontic appliances , removable OR orthodontic appliance design OR clear aligner OR invisalign AND marine environment OR terrestrial environment | 17 |
|  |  |  | orthodontic appliances , removable OR orthodontic appliance design OR clear aligner OR invisalign AND bisphenol OR bisphenol a | 92 |
|  |  |  | orthodontic appliances , removable OR orthodontic appliance design OR clear aligner OR invisalign AND toxicity | 115 |
|  |  |  | orthodontic appliances , removable OR orthodontic appliance design OR clear aligner OR invisalign AND bisphenol OR bisphenol a OR bisphenol a toxicity | 35 |
|  |  |  | orthodontic appliances , removable OR orthodontic appliance design OR clear aligner OR invisalign AND sustainable OR sustainability OR sustainable orthodontics | 101 |
|  |  |  | orthodontic appliances , removable OR orthodontic appliance design OR clear aligner OR invisalign AND recycling OR waste management | 37 |
|  |  |  | orthodontic appliances , removable OR orthodontic appliance design OR clear aligner OR invisalign AND health policy | 81 |
|  |  |  | orthodontic appliances , removable OR orthodontic appliance design OR clear aligner OR invisalign AND environment AND public health OR environmental impact AND health policy | 9 |
|  |  |  | orthodontic appliances , removable OR orthodontic appliance design OR clear aligner OR invisalign AND microplastics AND environment AND public health OR environmental impact AND health policy | 5 |
| Web of Science | All Fields | December 2025 | ((((ALL=(orthodontic aligner)) OR ALL=(clear aligner)) OR ALL=(clear aligner therapy)) OR ALL=(invisalign)) AND ALL=(microplastic) | 6 |
|  |  |  | ((((ALL=(orthodontic aligner)) OR ALL=(clear aligner)) OR ALL=(clear aligner therapy)) OR ALL=(invisalign)) AND ALL=(microplastics) | 7 |
|  |  |  | ((((ALL=(orthodontic aligner)) OR ALL=(clear aligner)) OR ALL=(clear aligner therapy)) OR ALL=(invisalign)) AND ALL=(environmental impact) | 24 |
|  |  |  | ((((ALL=(orthodontic aligner)) OR ALL=(clear aligner)) OR ALL=(clear aligner therapy)) OR ALL=(invisalign)) AND ALL=(public health) | 29 |
|  |  |  | ((((ALL=(orthodontic aligner)) OR ALL=(clear aligner)) OR ALL=(clear aligner therapy)) OR ALL=(invisalign)) AND ALL=(marine environment) | 2 |
|  |  |  | ((((ALL=(orthodontic aligner)) OR ALL=(clear aligner)) OR ALL=(clear aligner therapy)) OR ALL=(invisalign)) AND ALL=(bisphenol A) | 22 |
|  |  |  | ((((ALL=(orthodontic aligner)) OR ALL=(clear aligner)) OR ALL=(clear aligner therapy)) OR ALL=(invisalign)) AND ALL=(toxicity) | 12 |
|  |  |  | ((((ALL=(orthodontic aligner)) OR ALL=(clear aligner)) OR ALL=(clear aligner therapy)) OR ALL=(invisalign)) AND ALL=(sustainability) | 6 |
|  |  |  | ((((ALL=(orthodontic aligner)) OR ALL=(clear aligner)) OR ALL=(clear aligner therapy)) OR ALL=(invisalign)) AND ALL=(environmental sustainability) | 4 |
|  |  |  | ((((ALL=(orthodontic aligner)) OR ALL=(clear aligner)) OR ALL=(clear aligner therapy)) OR ALL=(invisalign)) AND ALL=(recycling) | 5 |
|  |  |  | ((((ALL=(orthodontic aligner)) OR ALL=(clear aligner)) OR ALL=(clear aligner therapy)) OR ALL=(invisalign)) AND ALL=(waste management) | 2 |
|  |  |  | ((((ALL=(orthodontic aligner)) OR ALL=(clear aligner)) OR ALL=(clear aligner therapy)) OR ALL=(invisalign)) AND ALL=(waste disposal) | 5 |
|  |  |  | ((((ALL=(orthodontic aligner)) OR ALL=(clear aligner)) OR ALL=(clear aligner therapy)) OR ALL=(invisalign)) AND ALL=(policy) | 17 |
|  |  |  | ((((ALL=(orthodontic aligner)) OR ALL=(clear aligner)) OR ALL=(clear aligner therapy)) OR ALL=(invisalign)) AND ALL=(health care policy) | 4 |

**PubMed Total Results: 244**

**Embase Total Results: 113**

**Scopus Total Results: 690**

**Web of Science Total Results: 145**
