# Supplementary material for: The Environmental Burden and Sustainability Challenges of Clear Aligners: Implications for Public Health, Practice, Policy, and Future Research—A Scoping Review
Source: Int J Dent. 2026 Aug 30;2026:2681058. doi: 10.1155/ijod/2681058 (PMC13526242; doi:10.1155/ijod/2681058)
Supplement: Supplementary file 2 [file IJOD-2026-2681058-s002.docx]

**Supplementary file 2: Characteristics of sources of evidence (n=61)**

| **Author, year,**  **Country** | **Study design or type of article** | **Aims and objectives** | **Methodology** | **Type of clear aligner material or system examined** | **Environmental or Public health impact indicators studied** | **Public health/ policy/ regulatory/ governance issues addressed** | **Key findings** | **Limitations or research gaps identified** |
| --- | --- | --- | --- | --- | --- | --- | --- | --- |
| Eliades et al. 2009 [19], Greece | In vitro study | To investigate in vitro cytotoxic and estrogenic properties of Invisalign appliances. | Immersion in saline for 2 months; eluents tested on Human Gingival Fibroblasts (MTT) and MCF-7 breast cancer cells. | Invisalign | Chemical leaching (BPA and residual monomers). | Hormonal endocrine disruptors; safety standards for BPA exposure. | No evidence of cytotoxicity on HGF and no stimulation of MCF-7 cell proliferation (estrogenic activity) was found. | Laboratory aging may not fully simulate aggressive intraoral factors like temperature shocks. |
| Martina et al. 2019 [20], Italy | In vitro study | To investigate in-vitro cytotoxicity of aligner materials on human gingival fibroblasts. | Four aligner materials were immersed in medium for 14 days and then MTT assay was performed. | Duran, Zendura, Biolon and SmartTrack | Cytotoxicity was studied. | - | All materials showed slight cytotoxicity, with Biolon the most. Thermoformed materials had higher toxicity than non-thermoformed. | Intraoral conditions cannot be completely simulated in in-vitro studies. |
| Nakano et al. 2019 [21], Japan | Technical Development | To develop biologically compatible resins with optimal safety for direct 3D printing of orthodontic aligners. | Development of acrylic-epoxy hybrid resins followed by evaluation of cytotoxicity, proliferation, and mechanical testing. | Direct 3D-printed aligners (Acrylic-epoxy hybrid light-curing resin). | Chemical leaching (aiming for BPA-free resin to avoid biohazards). | - | Successfully produced BPA-free biocompatible resins requiring improvement in mechanical strength and flexibility. | Mechanical properties need improvement for clinical use. |
| Iliadi et al. 2020 [22] | Systematic Review and Meta-Analysis | To appraise evidence on estrogenic/cytotoxic effects and BPA leaching from thermoplastic aligners. | 5 articles in qualitative synthesis and 2 in quantitative analysis. | Various thermoplastic aligners and retainers (Invisalign, Vivera, Essix). | BPA leaching | - | In vitro studies found no induction of MCF-7 cancer cell proliferation. One clinical trial found increased salivary BPA levels compared to Hawley retainers. | Methodological limitations in existing RCTs, limited number of studies and lab conditions cannot capture mastication stress. |
| Alhendi et al. 2022 [23], Saudi Arabia | In vitro study | To evaluate and compare the cytotoxicity of four different clear aligner systems. | Aligners were immersed in saline for 1 month and then eluents applied to gingival fibroblasts for 48 hours; MTT assay. | Four Aligner systems manufactured by thermoforming were studied, namely, Invisalign, Eon, Clarity, and SureSmile. | Chemical leaching of residual monomers. | Biocompatibility and adverse biological reactions to unpolymerized monomers. | All tested systems displayed slight to moderate toxicity, which generally increased with concentration. | Future studies needed in clinical situations to understand toxicity intraorally. |
| Alhendi et al. 2022 [24], Saudi Arabia | In vitro Study | To investigate and compare the leaching of four clear aligner systems. | Gas chromatography- mass spectrometry analysis of substances leached into ethanol/water solutions after two weeks. | Invisalign (SmartTrack), Eon (Polyurethane), SureSmile (Essix plastic) and Clarity were compared . | Leaching of 11 chemical compounds at higher concentrations of alcohol. No detectable BPA release in any system. | - | Invisalign leached the fewest chemicals (only Benzene). Eon leached the most (7 compounds). Alcohol concentration must be >50% for significant leaching. | In vitro conditions do not fully replicate intraoral factors like mechanical abrasion, pH variation, and enzymatic activity. |
| Francisco et al. 2022 [25], Portugal | Systematic Review | To analyze the release of toxic substances from 3D resins used in orthodontics and their systemic effects. | Systematic review of 14 articles using PRISMA, SYRCLE and Cochrane RoB tools. | 3D printed resins (SLA/DLP) and thermoformed resins. | Release of MMA, TEGDMA, HEMA, and Bis-GMA and leaching of metal trace elements (Al, Ni, Zn, Sn) in polymeric aligners. | - | 3D printed aligners may exhibit higher cytotoxicity than thermoformed resins if lacking final surface treatment. Metals detected did not exceed toxic levels. | Scarcity of clinical data, lack of sample randomization, inconsistent intervention protocols. |
| Frietas 2022 [26] | Editorial | To discuss environmental contamination caused by aligners and the role of orthodontists in mitigation. | - | Synthetic thermoplastic polymers. | Environmental contamination due to petroleum-derived hydrocarbons; leaching of BPA; toxicity of cyanides released during thermal degradation. | - | Classification as contaminated medical waste in the US and chances of cross-infection risk from improper disposal. Ethical responsibility of dental professionals. | Lack of awareness regarding correct disposal among the orthodontic community. |
| Gupta et al. 2022 [27], India | Narrative Review | To provide standard operating procedures for discarding used aligners to reduce biohazard and environmental impact. | Development of Standing operating procedures from literature. | Thermoplastic aligners- Considered all non-biodegradable materials | - | Management of Aligners as Biomedical Waste. | Since aligners are highly resistant to decomposition, the review highlighted the need for standard protocols for disposal as part of biomedical waste.. | - |
| Ozkan et al. 2022 [28], Turkey | In vitro study | To evaluate the in vitro cytotoxic effects of six clear aligner materials on human gingival fibroblasts. | Samples incubated in saline for 8 weeks at 37°C, followed by MTT analysis. | Invisalign SmartTrack, Duran, Zendura, Zendura-Flx, Taglus, and Essix C+. | Cytotoxicity was studied. | - | Zendura and SmartTrack showed mild cytotoxicity while Duran and Taglus showed no cytotoxicity. | In vitro conditions cannot simulate intraoral aging. |
| Peter et al. 2022 [8], India | Short Communication | To discuss rationale of an ongoing study. | Comparing plastic load compared to conventional fixed appliance therapy. | All clear aligner materials | Ways to minimize environmental impact is discussed | Uptake policy had been suggested for used aligners from the manufacturer’s side. | Solutions to reduce environmental impact suggested such as alternate modalities for orthodontic treatment, research into safer plastics and non-plastic materials and aligner uptake policy by manufacturers. | - |
| Pratsinis et al. 2022 [29] | In vitro study | To assess cytotoxicity and estrogenicity of novel 3D-printed aligners. | Aligners aged in deionized water for 14 days; eluents tested on HGF (MTT, ROS assays) and MCF-7 cells. | Direct 3D-printed aligners (Tera Harz TC85A resin; vinyl-ester urethane-based). | Chemical leaching from aligners | - | 3D-printed aligner eluates were not cytotoxic and showed no estrogenic activity. | The study does not account for complex intra oral conditions such as chewing forces or oral microbiota. |
| Bichu et al. 2023 [6] | Narrative Review | To review the materials used for aligner fabrication. | Comprehensive review of historical evolution, mechanical/thermal properties, clinical performance comparison and environmental burden of aligners. | PET, PETG, TPU, PP, PC, EVA, PVC, 3D resins (TC-85). | Large quantities of aligner waste leading to carbon emission and nanoplastics release. Waste disposal strategies currently adopted by few countries discussed. | Various strategies for reducing environmental impact discussed, like, biodegradable materials, newer 3D printed aligners, shape memory polymers etc. | 3D direct printing eliminates errors from thermoforming and generates less waste. But toxicity needs to be studied. | Paucity of evidence on toxicity from 3D resins, newer materials. |
| Caelli et al. 2023 [30], Italy | Life Cycle Assessment | To quantitatively compare the sustainability of thermoforming versus direct 3D printing technologies for clear aligner production. | Cradle-to-gate LCA using Open LCA software and Ecoinvent database to assess environmental impact categories for a functional unit of 40 aligners. | PETG and aliphatic vinyl ester-urethane polymer (direct 3D printing/DLP). | Considers environmental impact due to climate change, energy resources, acidification, eutrophication, water use, and raw material waste generation. | -. | Direct 3D printing (DLP) significantly reduces environmental impact by eliminating 3D-printed physical models, lowering energy consumption, and reducing water usage. | Unknown proprietary material compositions, limited chemical data in LCA databases and lack of primary data on use and disposal phases. |
| Gandhi & Veerasekaran 2023 [31], India | Narrative review | To review disposal methods, environmental hazards, material properties, and the need for correct disposal protocols. | Literature review of material properties, clinical concerns, and recycling strategies. | PET-G, TPU, PVC, Polysulfone, and Polycarbonate. | Waste generation and chemical leaching of BPA . | - | Environmental impact of clear aligners needs to be considered, BPA release could be mitigated by rinsing before use and recycling should be considered involving all stakeholders. | Need for standardized usage and disposal recommendations and insufficient awareness among orthodontists and patients. |
| Mendes Ribeiro et al. 2023 [32], Brazil | Narrative review | To compile scientific evidence regarding the effects and limitations of aligners. | Evidence retrieved from six electronic databases presented through a question-and-answer format. | All aligner materials | BPA and nanoplastics release. Aligners identified as environmental hazards/medical waste | - | BPA release remains questionable but possible. Clinical experience currently outweighs robust scientific evidence. | Urgent need for well-designed RCTs while material evolution outpaces research results. |
| Özkan and Gök 2023 [33], Turkey | In-vitro study | To evaluate BPA, BPF, and BPS release of six different clear aligner materials. | Samples kept in saline for 8 weeks at 37°C and LC-MS/MS analysis to detect trace elements. | Invisalign SmartTrack, Duran, Zendura A, Zendura Flx, Taglus Premium, and Essix C+. | Chemical leaching: BPA and BPS release. | - | All leached levels were below reference daily intake doses; SmartTrack had the highest release of BPA and BPS. | Experimental settings lack intraoral conditions such as mechanical and chemical abrasion and salivary enzymes. and also there is need for studying cumulative effects over time. |
| Peter et al. 2023 [34], India | Systematic Review | To synthesize evidence on the release of BPA from thermoplastic clear aligner materials. | Systematic search of 6 databases and 6 studies were included. | Invisalign (Polyurethane), Essix ACE (PET-G), SmileDirect Club. | BPA leaching was studied | - | Conflicting evidence with one RCT showed significant leaching, while four in vitro studies found no traces. But the level of evidence was low to very low. | Moderate to high risk of bias, low to very low level of evidence and secret compositions maintained by manufacturers. |
| Quinzi et al. 2023 [10] | In vitro study | To evaluate the potential detachment of microplastics (MPs) from clear aligners due to mechanical friction in artificial saliva. | Seven brands of aligners were tested in artificial saliva for 7 days with 5h/day stirring to simulate chewing friction. Analysis was done with. Raman Microspectroscopy (RMS) and SEM. | PET and Polyurethane aligners. Thermoforming vs. direct 3D printing. | Release of secondary microplastics (3μm to 50 μm). | Systemic health risks from microplastic ingestion | Confirmed MP detachment across brands. 3D-printed aligners showed significantly lower MP release than thermoformed brands. Most particles were 5–20 μm. | Difficulty in perfectly replicating in vivo friction and need for studies at different wearing times. |
| Willi et al. 2023 [35] | In vitro study | To assess molecular structure and water-leaching of targeted compounds from 3D-printed aligners. | 3D printing (Tera Harz TC85A) followed by ATR-FTIR for degree of conversion (DC) and LC-MS/MS for UDMA and BPA after 1 week. | 3D-printed aligner resin (Tera Harz TC85A). | Chemical leaching: UDMA detected and BPA was not detectable. | Potential health hazards of UDMA. | DC was estimated at 83% and high variability in UDMA leaching suggests non-standardized process. | In vitro setup cannot simulate intraoral environment. |
| Yazdi et al. 2023 [36], Iran | Systematic review | To summarize the biocompatibility and safety of clear aligners, specifically focusing on BPA release and cytotoxicity. | Systematic review of 16 articles (1 RCT and 15 in vitro studies) searched across Web of Science, PubMed, and Scopus. | Invisalign (SmartTrack), Vivera, Essix (A+, ACE), Biocryl, and generic thermoplastic sheets. | Chemical leaching of BPA and monomers (MMA); one clinical trial showed very high BPA levels in saliva. | Potential safety threats to children/preadolescents from endocrine-disrupting chemicals. | In vitro studies show negligible BPA leaching, but a clinical trial showed significant salivary BPA levels. Most materials show slight cytotoxicity. | Inconsistency between in vitro and in vivo results; lack of standard risk-of-bias tools for in vitro studies. |
| Alkhamees 2024 [37], Saudi Arabia | Narrative Review | To review 3D printed aligners. | Comprehensive review of manufacturing protocols (thermoforming vs. 3D printing). | Thermoformed PETG/PU and 3D printed photocurable resins (e.g., Tera Harz TC-85). | Plastic waste burden, generation of non-recyclable dental models; 3D printing reduces refuse and carbon emissions vs. thermoforming. | Environmental contamination through microplastic and highlighting need for recyclable materials. | 3D printed aligners offer higher precision. Shape Memory Polymers can reduce the number of aligners needed. Graphy’s resin is reported as biocompatible and eco-friendly. | Limited evidence-based studies on clinical effectiveness and long-term toxicity of printable resins; lack of clinical performance comparisons. |
| Azhagudurai et al. 2024 [38], India | Ex Vivo Study | To detect and quantify BPA leaching in salivary samples of patients wearing four aligner brands. | 20 volunteers wore aligners (PETG); saliva collected at days 0, 1, 5, and 7; analysis via LC-MS. | Monoflex, Erkodur, Leone, and Duran (all PETG). | Chemical leaching (BPA). | BPA as an endocrine disruptor; acceptable daily intake (ADI) doses by FDA. | Significant leaching occurred only at 24 hours (Day 1). Duran released the most BPA (1.24 ppm). | Small sample size; variation among non-PETG compositions not explored; attachments not included. Clinical trials should explore this further. |
| Bouchema et al. 2024 [39], France | In-vitro study | To evaluate material properties of ClearCorrect aligners before and after intraoral use, focusing on leachables and performance. | Analysis of used (14 days) and unused aligners. | ClearCorrect aligners (Three-layer structure: outer PETG / inner PU layers). | Release of organic compounds (oligomers) and trace elements (Al, Si, Cu, Zn, Mn, Fe, Mg) while no BPA release detected. | Patient safety regarding long-term ingestion of leachables. | Intraoral aging increases surface roughness. Trace elements detected in unused samples, decreasing after use. | Study focused on a single patient and lack of long-term health impact assessments for cumulative contaminant exposure. |
| Krishnan 2024 [40] | Editorial | To raise awareness regarding microplastic release from aligners and potential health/environmental risks. | Bring to light microplastic detachment from aligners. | PETG and TPU (petroleum-based polymers). | Detachment of secondary microplastics due to friction. | Health hazards due to microplastic release. | Mechanical friction causes microplastic detachment from all major brands. | Gap in knowledge regarding leached components and overlooking harms due to CAT popularity. |
| Lo et al. 2024 [41], Taiwan | In vitro study | To assess the cytotoxicity of original and thermoformed thermoplastic aligner materials on Human PDL cells. | Six brands soaked in medium for 7/14 days; Human PDL cell viability analyzed via MTT assay. | PETG (Duran, Essix, Leone), TPU (Maxflex, Zendura), and PET (Keystone). | Chemical leaching. | - | Most materials maintained >70% viability at 7 days (thermoformed better than non-thermoformed) while thermoformed PETG showed lower survival rates at 14 days than non-thermoformed versions. | Did not specifically measure concentration of released monomers and there is a need for long-term research. |
| Macri et al. 2024 [2], Italy | Narrative Review | To examine environmental implications of CAT and explore sustainability strategies. | Bibliographic research using PubMed and Google Scholar. | PET, PETG, TPU, and 3D direct printing resins (Graphy, 3Dresyns). | Sustainable waste management methods discussed. | - | Numerous strategies for better environmental management of clear aligners has been discussed like biodegradation, biodegradable polymers, treatment strategies, manufacturing, patient education etc. | - |
| Palmieri et al. 2024 [42], Italy | In-vitro study | To evaluate 4D printing for sustainable orthodontic aligners with a reduced carbon footprint (ClearX system). | Morphological, optical, and mechanical evaluation of aligners before/after thermal reshaping in boiling water for different time periods. | ClearX 4D-printed aligners (Copolyester-polyurethane shape-memory polymer). | It has been suggested that carbon footprint reduction by half (1.8 kg CO2 vs typical 4 kg) could be possible by reducing the number of aligners needed via reshaping. | - | 4D aligners retain structural properties after reshaping. Temporary transparency loss is reversible and caused by transient swelling, not degradation. | Evaluated only one 4D system (ClearX) and laboratory tests may not reflect complex in vivo mechanical stresses. |
| Panayi et al. 2024 [7] | Narrative Review | To explore concerns arising from microplastics and nanoplastics in aligner orthodontics. | Review of literature on polymer degradation, MNP uptake mechanisms, and health implications | All aligner materials | Microplastic release, leaching of BPA and environmental waste generated from nonbiodegradable aligners. | Impact of released micro and nano plastics on gut health, respiratory and cardiovascular health. | Aligners release microplastics due to friction and aging and MNPs can cross blood-brain and placental barriers. | - |
| Tunca 2024 [43] | Letter to Editor | Discusses about the importance of recycling and disposal of aligners. | - | Clear Aligners in general. | - | - | Acknowledges differences in waste legislation across manufacturing countries and the need to involve all stakeholders in recycling and proper waste disposal. | - |
| Turkalj et al. 2024 [44], Belgium | In vitro study | To evaluate cytotoxic effects of thermoplastic materials used for orthodontic appliances. | XTT and LDH assays on primary human gingival fibroblasts and cell cycle analysis via flow cytometry of 24h and 14-day extracts. | Essix (C+, Plus, Tray Rite), VacuFormer (Bleach Heavy, Splint, X-Heavy), Invisalign. | Chemical Leaching | - | Extracts from all materials did not evoke cytotoxicity under the experimental conditions. | Information regarding composition from manufacturers is often incomplete and hinders identification of eluted compounds. |
| Veseli et al. 2024 [45] | Editorial | It brings attention potential hazards of clear aligners. | - | Clear Aligners in general. | Micro and Nanoplastic release from clear aligners. | - | Highlights the health effects of microplastic release from aligners and poses solutions in the form involving all stakeholders. | - |
| Bandić et al. 2025 [46], Croatia | In vitro study | To evaluate the cytotoxicity of different thermoplastic aligner materials and the impact of the thermoforming process. | Four materials tested using CCK-8 assay on human oral fibroblast cells after 14 days incubation in artificial saliva. | TPU (Zendura A), double-layered PETG/TPU (Erkoloc-pro), and three-layered copolyester/elastomer (Zendura FLX, CA Pro +). |  | Biocompatibility and patient safety are a concern. | Chemical leaching affecting cell viability and cytotoxicity threshold (70%) reached by certain concentrations after 72h. Thermoforming itself did not significantly increase the cytotoxicity of the materials. | Lack of data on proprietary material compositions and in vitro conditions do not fully replicate mastication; need for intraoral studies. |
| Barile et al. 2025 [47], Italy | In vitro study | To study mechanical damage and microplastic release under cyclic compression simulating swallowing. | Cyclic compression in artificial saliva; inverted optical microscopy and MATLAB image processing. | Thermoformed PET-G. | Microplastic release where particles ranged from 685μm to 5097μm in surface area. | - | Released microplastics were large (> 20μm), likely to be excreted rather than absorbed, less likely to be cytotoxic. | The study was limited to one type of material. |
| Boonchanachai et al. 2025 [3] | Scoping Review | To synthesize evidence on the environmental impact of clear aligner therapy and evaluate sustainable strategies guided by UN SDGs. | Analysis of 27 studies using the 4R framework (Reduce, Reuse, Recycle, Rethink) aligned with UN Sustainable Development Goals. | All aligner materials | - | Looks into strategies for waste management and adoption of 4Rs. | Offers many sustainable solutions to reduce clear aligner impact like digital workflows, staged production and 3D-printed aligners. | Absence of comprehensive lifecycle analyses; limited region-specific data from emerging markets; lack of formal quality assessment of sources. |
| Bor et al. 2025 [48], Turkey | In vitro study | To compare the cytotoxicity of two 3D-printed aligner resins and evaluate post-curing protocols. | Samples printed with Ackuretta and cytotoxicity was tested via XTT and RTCA on human gingival fibroblast cells. | 3D-printed resins: Tera Harz TC-85 DAC and Clear-A. | Chemical leaching of residual monomers. | Biocompatibility and regulatory approval of 3D printed resins is required. | All tested 3D-printed resins and post-curing protocols exhibited biocompatibility (>70% viability). | Sample dimensions did not represent full clinical surface area and chemical composition of extracts was not evaluated. |
| Camcı and Büyükbayraktar (2025) [5], Turkey | Narrative Review | To assess if aligners pose a long-term environmental threat and propose solutions (4Rs). | Review of global plastic waste statistics, aligner production estimates, and sustainability reports. | Polyester, Polyurethane, Polypropylene; 3D-printed resins; 4D aligners. | Reports of massive waste generation (est. 1456 tons used aligners + 7000-8000 tons scrap). | - | Proposes many solutions to environmental threat of aligners like 3D printing reduces waste by eliminating models, 4D aligners reduce tray numbers and need for recycling programs. | Lack of studies comparing decomposition times of different materials. |
| Dantagnan et al. 2025 [49], France | Systematic review | To investigate residual monomer leaching and toxic effects specifically from direct printed clear aligners. | Systematic review following PRISMA guidelines; 9 in vitro studies included; risk of bias assessed via QUIN tool. | 3D printed resins: Dental LT (Formlabs), TC-85 DAC (Graphy), E-Guard, Freeprint splint 2.0. | Chemical leaching: High level of UDMA release detected; HEMA and EGDMA release from some resins; no traces of BPA leaching. | Safety concerns for adolescents in puberty regarding active monomers like UDMA; necessity of strict post-curing. | 3D printed aligners exhibit higher cytotoxicity than thermoformed ones and UDMA is the primary monomer released. | No consensus on absence of cytotoxic effects and behavior of 3D resins in vivo remains undetermined. |
| De Stefano et al. 2025 [50], Italy | Systematic review | To investigate if clear aligners release microplastics and identify potential implications for human health. | Systematic literature search in PubMed, Scopus, and Web of Science; 11 articles were included. | Thermoformed systems (Invisalign/TPU, PETG) and directly printed aligners (DPA). | Release of secondary microplastics; particle sizes ranging from 5 to 20 μm detected following simulated use in one study. | Ill-effects of microplastics. | Gradual increase in surface roughness indicates degradation. Microplastic dispersion occurs during use and smaller particles pose higher risks. | Support mainly from in vitro studies while proprietary material compositions remain unknown. |
| Delgado et al. 2025 [51], Spain | Narrative Review | To summarize trends in clear aligners. | Review of commercial thermoplastics (PETG, TPU, PC, etc.) focusing on optical, mechanical, biocompatibility properties and recycling of aligners. | PETG, PET, hTPU, PC, EVA, PP, PVC, and 3D resins (TC-85, Dental LT). | Waste from thermoforming molds, carbon footprint and leachate emissions. | - | Summarizes information on aligners regarding properties including cytotoxicity and environmental impact. | - |
| Dhayananth et al. 2025 [52], India | In vitro study | To assess and compare polymer leaching of various aligner materials using potassium permanganate (KMnO4). | Powdered materials were immersed in KMnO4 and leaching quantified via colorimetric analysis. | PETG (Duran), Photopolymer (NextDent), Polyurethane (Zendura), and SmartTrack (Invisalign). | Polymer leaching (unreacted monomers, additives, and by-products). | Biocompatibility and systemic safety; toxicity of leached compounds such as BPA and phthalates. | All materials exhibited leaching; PETG showed the highest leaching potential followed by Photopolymer, Polyurethane and SmartTrack. | Limitations in the method adopted in this study. Specific chemical compounds leaching from aligners cannot be identified with this method. |
| Ferreira et al. 2025 [53], Portugal | Systematic Review | To understand the impact of clear aligner on the oral cavity of patients. | Systematic search across PubMed, Scopus, Cochrane. Included 7 articles (in vitro and in vivo). | Polyurethane and PETG aligners; mention of fluoride-coated and 3D-printed models. | Chemical leaching of BPA and monomers; bacterial accumulation; inflammatory responses. | - | Aligners are generally safe but release traces of BPA. Thermoforming significantly increases cytotoxicity in some materials (e.g., TPU). | Need for long-term clinical trials on systemic effects; variations in oral pH and hygiene influence leaching. |
| Goracci et al. 2025 [54] | Critical Review | To revise evidence on the biological properties of directly 3D-printed aligners (DPAs). | 11 studies were included focussing on marketed resins: Tera Harz TC-85 DAC, TA-28, and Clear-A. | Directly 3D-printed aligners (DPAs) vs. thermoformed systems. | UDMA leaching and absence of BPA in tested 3D resins. | REACH and Medical Device Regulations and need for transparency of chemical patents. | TC-85 DAC resin is consistently non-cytotoxic and non-estrogenic if post-cured in nitrogen (oxygen-free atmosphere). | Limited data for most marketed resins, lack of standardization in protocols and scarce long-term in vivo data. |
| Jeyachandran and Aman 2025 [55] | Narrative Review | To assess microbial-mediated biodegradation of PET used in aligners to mitigate environmental plastic waste. | Literature review of analytical methodologies, molecular tools, and biotechnological strategies. | Polyethylene terephthalate (PET) and PET-based copolymers. | Sustainable Waste management with biodegradation using microbial consortia. | - | Microbial consortia are more efficient than single microbes at breaking down PET and can be a sustainable solution for the environmental hazard of aligners. | Slow degradation rates, complexity of dental material additives/plasticizers and challenges in scaling up such processes. |
| Lan et al. 2025 [56], China | In vitro study | To explore and optimize alkaline hydrolysis as a method for chemical recycling of clear aligners. | Alkaline hydrolysis of aligner powder using NaOH and ethanol-water mixtures. | Angel Aligner (Polyurethane/PETG-based thermoformed material). | Recycling yield of 4,4′-methylenedianiline (MDA) which can be reutilized and resulting in reduction of environmental emissions. | Environmental protection and waste management of non-biodegradable plastic medical devices. | Clear aligners can be effectively recycled into valuable monomers (MDA) using alkaline hydrolysis. Optimized conditions: 130°C, 5 wt% NaOH. | Proprietary compositions vary, scaling up to industrial production and 3D-printed materials may require different methods. |
| Lea et al. 2025 [57], Slovenia | Systematic Review | To summarize in-vitro evidence on the biocompatibility of clear aligners and thermoplastic retainers. | Systematic search of PubMed, Cochrane, and Google Scholar and included 17 in vitro studies. | Thermoformed PETG (Essix ACE, Duran), multilayer PU (SmartTrack), and 3D printed resins (Graphy TC-85DAC). | BPA undetectable or low ng/mL while UDMA leached from printed resins and microplastic detachment was noted in mechanical wear contexts. | - | Biocompatibility is generally favorable and direct printed resin safety depends heavily on post-processing. | Lack of studies pairing microplastic release with biological endpoints and heterogeneity in extraction conditions among studies. |
| Lorusso et al. 2025 [58], Italy | Systematic Review | To assess the cytotoxicity of 3D-printed aligners. | Electronic search up to Feb 2025 and five in vitro studies were included. | 3D-printed resins (Graphy TC-85 and TC-28) and Form Cure. | Cytotoxicity due to release of unused monomers. | - | Cytotoxicity is influenced by post-curing duration and the curing systems used. | Lack of randomized clinical trials and methodological variability across in vitro studies. |
| Mallik et al. 2025 [11], India | Umbrella review | To examine the biological effects of BPA leaching from clear aligner materials and focus on endocrine disruption risks. | Systematic search followed by quality assessment using AMSTAR-2. | Various thermoformed systems (Invisalign, ClearCorrect, Duran, Zendura). | BPA leaching. SmartTrack polyurethane exhibited the highest release of these chemicals. | Public health risks such as endocrine disruption, reproductive health, cancer risk, from BPA release | In vitro studies show minimal cytotoxicity but clinical trials detect higher BPA levels due to dynamic oral environments. | Inconsistency between in vitro and clinical results. Long term clinical studies needed. |
| Nunes et al. 2025 [59], Portugal | In vitro study | To evaluate and compare the cytotoxic effects of five common aligner brands on various cell lines. | Cell lines (MRC-5, 3T3-L1, Vero) exposed to conditioned media; MTT assay for viability; flow cytometry for cell cycle. | ClearCorrect, Invisalign (SmartTrack), Spark (TruGEN), SureSmile, Essix PLUS. | Chemical leaching of residual monomers, plasticizers, or stabilizers; cytotoxic impact. | Lack of transparency from manufacturers regarding material chemical composition. | Invisalign and Spark showed highest cytotoxicity among the brands (viability <70%). Thermoforming reduced the cytotoxicity of Essix PLUS compared to its non-thermoformed state. | Use of non-oral cell lines, need to chemically analyse leaching compounds, lack of chronic toxicity data and need for in-vivo/clinical studies. |
| Nunes et al. 2025 [60], Portugal | Umbrella Review | To determine if clear aligners promote cytotoxic and estrogenic effects. | Systematic search included 4 systematic reviews. | PVC, PU, PET, PETG, and 3D printed resins. | Leaching of monomers (BPA). | - | Aligners generally lack significant estrogenic/cytotoxic capacity in vitro. BPA leaching is usually below toxic thresholds. 3D printed aligners may exhibit higher cytotoxicity. | Included SRs were of low quality and high risk of bias, lack of clinical studies evaluating safety and short follow-up periods. |
| Pendse et al. 2025 [61], India | In vitro study | To investigate cytotoxic effects of four aligner materials on human gingival mesenchymal stem cells. | MTT assay on HGFs harvested at three time points. | TAC (PETG), Flash (PET), Taglus Premium (PET), Invisalign (SmartTrack - TPU). | Cytotoxicity was studied. | - | All materials showed no cytotoxicity. TAC showed the most cell growth, but differences were not statistically significant. | In vitro methods cannot fully replicate intraoral environment. |
| Peter et al. 2025 [62], India | In vitro study | To analyze smoke composition from incinerating common aligner materials (PET-G and Polyurethane). | Thermoforming, shredding, and open-fire combustion of samples, smoke collection on quartz fiber filters followed by GC-MS analysis. | PETG-based (Duran) and Polyurethane-based (Zendura). | Air pollution: 20 compounds identified per material; PETG smoke contained Benzene; Polyurethane smoke contained Tetrahydrofuran. | - | Incineration of aligners releases carcinogenic and toxic fumes. | Study focused only on two materials and toxicologic effects of many identified compounds require further investigation. |
| Tania et al. 2025 [63], India | In vitro study | To determine release of BPA from five different orthodontic aligners. | Abraded samples immersed in artificial saliva and subjected to thermal shock followed by analysis with GC-MS. | Dentcare, Invisalign, SD Align, Smile Aligner, and TAC Aligners (Polyurethane). | Chemical leaching (BPA, Diethyl phthalate/DEP). | - | BPA was not detected in any group; however, Diethyl phthalate (DEP) was identified, raising alternative toxicity concerns. | Need for meticulously designed in-vivo studies. |
| Teramoto-Iida et al. 2025 [64], Mexico | In vitro study | To develop bioactive orthodontic aligners by combining 3D-printed shape-memory resin with antimicrobial ZnO nanoparticles. | Green synthesis of ZnO NPs; coating 3D-printed discs (TC-85DAC) with 10–30% ZnO NPs and antibacterial testing and hGF assays. | Direct 3D-printed shape-memory resin (TC-85DAC) with coating. | Development of biocompatible antibacterial material | - | ZnO NP coatings (30%) significantly enhanced antibacterial activity and cell viability. | White coating may compromise esthetics and need for durability studies under oral conditions. |
| Torkomian et al. 2025 [65], Spain | Systematic Review | To assess differences between traditional thermoformed (TFA) and emerging 3D-printed aligners (DPA) regarding properties and sustainability. | Systematic review of 37 articles. | Thermoformed PETG (Duran, Erkodur, Invisalign) and 3D-printed TC-85 DAC resin. | 3D printing (DLP) seems to reduce environmental impact compared to thermoforming. | - | DPA showed higher accuracy and trueness. DPA (TC-85) has shape memory properties. UDMA detected in TC-85 leachates, but no BPA. | Limited studies on sustainability and biocompatibility of DPA, lack of RCTs AND absence of studies on aligner recyclability. |
| Umrai Shariff et al. 2025 [66] | Narrative Review | To evaluate the impact of microplastics (MPs) and nanoplastics on human health in clinical dentistry and orthodontics. | Literature retrieval from PubMed and Google Scholar (53 records; 11 selected for synthesis). No formal risk-of-bias evaluation. | PETG, Polypropylene, and Polyurethane-based multilayer copolymers. | Secondary MP detachment and release from clear aligners was discussed. | - | Clear aligners release MPs (5–20 μm) during routine wear. Mechanical friction and bruxism increase release. Smaller MPs (<5 μm) can cross membranes and gut epithelium. | Inconsistent testing protocols, reliance on visual detection, lack of in vivo clinical evidence on long-term biological effects and need for standardized testing. |
| Vitores-Calero et al. 2025 [67], Spain | Prospective Observational Study | To analyze BPA release in the saliva of patients wearing Invisalign vs. in-office thermoformed aligners. | 24 patients; saliva collected at T0, T1 (after attachments), T2 (after aligners), and T3 (1 week); analysis via HPLC-MS. | Invisalign (SmartTrack) vs. In-Office (Ortolan thermoforming sheets). | BPA release in saliva was studied in vivo. | - | Attachments are the primary source of immediate BPA increase and all levels remained below international safety limits. | Small sample size, short 1-week follow-up and no data on direct 3D printed aligners. |
| Zecca et al. 2025 [68], Italy Switzerland | In vitro study | To compare DPA and TFA regarding potential microplastic and nanoplastic release under simulated oral conditions. | Mechanical friction simulation (1kg force) in ultrapure water; analysis via OM, TEM, AFM, and gravimetric quantification. | DPA (Graphy Tera Harz TC-85-DAC) vs. TFA (Invisalign SmartTrack). | Studied plastic release from aligners | - | DPA generated significantly larger and more numerous plastic particles compared to TFA. | Absence of saliva might underestimate friction. Chemical composition of released particles was not assessed in the study. |
| Zecca et al. 2025 [69], Italy | In vitro study | To evaluate acid-induced degradation and elemental release from TFA and DPA in a simulated gastric environment. | Samples exposed to HCl (pH 2); surface analysis via AFM and elemental analysis via ICP-MS. | TFA (Invisalign SmartTrack) and DPA (Graphy TC-85DAC resin). | Elemental release in acidic environment like gastric acids. | Safety of ingested microplastics and nanoplastics and systemic distribution following gastric degradation. | DPA exhibited slower acid erosion than TFA. TFA released more Sn and Sb; DPA leachates were richer in Ca and Fe. | Simulated environment lacks gastric enzymes and peristalsis and the study also focused on only two specific brands. |
| Andrew Choi et al. 2026 [70], South Korea, USA | Experimental Study (In vitro/Clinical) | To evaluate how clinical use affects surface properties and cytocompatibility of 3D-printed aligners. | Collection of 11 used aligners (10 days), along with non used sampled exposed and not exposed to artificial saliva. SEM topography; MTT assay (L929 cells); qPCR for proinflammatory markers. | 3D-printed direct aligners (Graphy TC-85DAC). | Cytotoxic release and proinflammatory response in presensitized cells was studied. | - | Cytoxicity was not observed in any of the studied samples. Salivary exposure increases surface irregularities and used aligners exacerbated proinflammatory markers in presensitized cells. | Patients were not given any standardized wear instructions which might have influenced the results. Use of mouse fibroblasts instead of human oral cells and lack of masticatory force simulation in artificial saliva group. |
| Wendl et al. 2026 [71] | In vitro study | To clarify biocompatibility and leachable compounds of different aligner materials. | Headspace SPME-GC-MS for volatiles, GC-MS for BPA/BPS quantitation after 72-hour artificial saliva immersion. | CA Pro Clear, Erkodur-al (thermoformed), Graphy (3D printed TC-85 DAC), and SMP aligner. | Chemical leaching: no BPA detected; BPS found in Graphy-Aligner. | - | Direct printed aligners leached more compounds than thermoformed ones and most leaching occurs in the first 24 hours. | In vitro conditions don't reflect mechanical abrasion in oral cavity and cumulative effects remain under-researched. |
